# Redefining feasibility in clinical trials: Collaborative approaches for improved site selection

**DOI:** 10.1016/j.conctc.2024.101291

**Published:** 2024-05-31

**Authors:** Beau Bruneau, Kristin Surdam, Amy Bland, Amy Krueger, Andrew Wise, Ani Cotarlan, Asher Leviton, Elena Jouravleva, Grace Fitzgerald, Heather N. Frost, Honora F. Cutler, Joshua Buddle, Luis G. Diaz, Michele Cohen, Nancy A. Sacco, Ryan Washington, Susan Mauermann, Victor Chen, Andrea Bastek

**Affiliations:** a Florence Healthcare, USA; b KOL & Strategy, Florence Healthcare, USA; c Clinical Research, Baptist Health Little Rock, USA; d Bon Secours Mercy Health, USA; e Icahn School of Medicine at Mount Sinai, USA; f Corporate Quality and Education, Sarah Cannon Research Institute, USA; g Global Clinical Information Solutions, Information Management, Pfizer Research & Development, Pfizer, USA; h Clinical Research Operations, Drexel University, USA; i Clinical Operations, Flatiron Health, USA; j Regulatory, University of South Florida and Tampa General Hospital, USA; k Clinical and Site Development Ops, SiteBridge Research, Inc, USA; l Cancer Clinical Trials Office, SUNY Stony Brook Cancer Center, USA; m Medical College of Wisconsin, USA; n Clinical Trials Program, The Permanente Medical Group, USA; o Innovations, Florence Healthcare, USA; p Site Enablement League, USA; q Elevation GCP Consulting, LLC, USA; r Icahn School of Medicine at Mount Sinai, USA; s Icahn School of Medicine at Mount Sinai, USA

**Keywords:** Clinical trial operations, Clinical trial feasibility, Site feasibility

## Abstract

**Background:**

This Site Feasibility Task Force convened to assess the complex and burdensome process of site feasibility in clinical trials. The objective was to create mutual understanding of challenges and provide suggestions for improving collaboration among sponsors, contract research organizations (CROs), and sites.

**Methods:**

The task force was composed of representatives from sponsors, CROs and sites (43 % Sites, 20 % Site Networks, 10 % Small/mid-size sponsors, 10 % Small/mid-size CROs, 10 % Large sponsors, 7 % Large CROs). The group collaborated to define the scope of the problem, identify challenges in the current process, and provide suggestions for improving the process.

**Results:**

The group found there is a need for better differentiation between the three main stages of feasibility, and the four sub-phases of Site Feasibility. The discussion brought to light emerging trends like early initiation of Site Feasibility and premature engagement of sites by CROs. To fully explain these challenges, the group analyzed the current practices and documented their downstream impact on clinical trial execution for all stakeholders. A list of best practices emerged naturally from this analysis. These findings are aggregated into short and actionable best practice guides.

**Conclusion:**

The task force suggests practical changes for the feasibility process and raises awareness of emerging trends and their associated risks. This awareness can begin to drive change in the site feasibility process, although industry-wide transformation will require new levels of collaboration, data standardization and automation tools. The potential benefits of evolving this process are significant and meaningful for more efficient and successful clinical trials.

## 1 Introduction

### 1.1 The task force

The Site Enablement League (SEL) is focused on streamlining processes, implementing advanced technologies, and fostering effective communication among all industry stakeholders to create a more agile and innovative clinical research environment, leading to faster development of life-saving treatments. The SEL Task Force on Feasibility was convened to bring focus and collaboration to improving the process of site feasibility. The group was composed of representatives from sponsors, CROs and sites (43 % Sites, 20 % Site Networks, 10 % Small/mid-size sponsors, 10 % Small/mid-size CROs, 10 % Large sponsors, 7 % Large CROs). The broad representation of the SEL Task Force increases the group's potential to identify newly emerging challenges, increase awareness of the challenges each party faces, and formulate practical recommendations for improvement.

### 1.2 Defining the scope of work

The SEL Task Force convened to bring focus and collaboration to improving the process of site feasibility. When discussing feasibility, it is important to first align on the definition and scope of the process. The concept of “feasibility” in clinical trials is multifaceted, encompassing three stages of the clinical trial process, Program Feasibility, Study/Protocol Feasibility and Site Feasibility [1], defined here.

● **Program Feasibility** - This involves assessing disease prevalence, competitive landscape, regulatory norms, and geographical aspects to craft a trial program.
● **Study/Protocol Feasibility** - This includes evaluating clinical, technical, regulatory, geographic, and operational components of a particular protocol to ensure optimal project completion concerning timelines, targets, and costs.
● **Site Feasibility** - This entails identifying and assessing potential sites for a specific study. The process may also be called a Feasibility Assessment (FA) [2], Site Qualification or Site Selection. The process may include a survey or feasibility questionnaire (FQ) as well as a site visit (known as pre-selection visit (PSV), pre-study site visit (PSSV), or site qualification visit (SQV)). In this work we will refer to the process as Site Feasibility.

The SEL Task Force determined that the site feasibility process can be further segmented into four areas.

○ **Site Profile information** - basic site information such as address, specialty areas, number of physicians, startup process, review committees required, etc.
○ **Site Capability information** - what equipment does the site have, what lab tests can be performed, etc.
○ **Site Performance information** - past enrollment performance to target, inspection findings, data quality metrics, etc.
○ **Specific Protocol assessments** - patient population estimates, referral patterns for the disease state, standard of care (SOC) details for the disease state in question, ability to conduct specialized procedures or tests, etc.

A limited number of industry professionals participate in all three stages, causing the term “feasibility” to hold a unique meaning to each individual involved in the clinical trials process. Thus, when working with a cross-functional, cross-organizational group, it becomes crucial to clarify the process under discussion. This work is focused specifically on Site Feasibility.

### 1.3 Prior efforts to improve site feasibility

The site feasibility process is critical for sponsors and CROs to select qualified sites (as required by federal regulations such as 21CFR312.53 and 21CFR812.43) and yet the process is plagued by excessive site burden, inefficiencies and lack of transparency [2,3]. These major challenges are well-recognized among sites and are often a topic of discussion among clinical researchers. The American Society of Cancer Oncology Task Force recently released survey results summarizing these commonly understood but poorly documented issues with site feasibility for oncology sites and highlighting the scale of the challenge [2]. The results offer a quantitative measure of the problem's scale and impact in oncology. The results are applicable across all therapeutic areas, and therefore the scale of the problem across the industry is estimated at $1.6B [4].

There have been efforts to improve the process over the last decade [[5], [6], [7], [8], [9], [10], [11]], generally falling into three categories: standardization of questionnaires, standardization of technology, or accreditation of research sites. However, none of the prior efforts have been widely adopted by industry stakeholders. The SEL Task Force discussed that a primary reason for the lack of success in prior efforts is the difficulty in driving collaboration and buy-in for a standardized solution across all stakeholder groups.

In spite of the lack of material progress in standardization, the ASCO paper still recommended that the industry should standardize the site feasibility process and questions. The Avoca Quality Consortium (AQC), in response to the call to action from ASCO, focused on the development of a new suite of site feasibility tools, including standard profiles, feasibility forms and checklists that are simple, effective, standardized with minor adaptability, and flexible enough for sites. Both AQC and ASCO acknowledge that standardization requires collaboration between all stakeholders, and lack of buy-in from sponsors and CROs is a major barrier to change. Although these new tools have the potential to be a catalyst for collaboration between stakeholders, they are not publicly accessible and require membership for usage, thereby reducing their potential influence across the industry.

The ASCO paper also recommends leveraging technology to improve site feasibility. However, technology as a solution to feasibility has been attempted without success or widespread adoption [2]. Transcelerate BioPharma created the Shared Investigator Platform along with 7 of the largest research sponsors in 2016, alleging “… sites will experience: increased efficiency and reduced administrative burden in clinical trial planning & conduct” [6]. Members of the SEL Task Force cited frustrations from using this technology due to lack of broad sponsor and CRO buy-in, redundancies in requests for data that had already been shared in the platform, inflexibility in workflows and lack of integrations to site systems, to name a few.

Finally, the effort to create a standard accreditation process for research sites with the goal of increasing the quality and efficiency of clinical trials [3,7] has also been unsuccessful. This idea relies on uniform sponsor recognition of accreditation as the main value proposition for sites to undergo the lengthy and expensive accreditation process and also requires an unbiased organization to develop and operationalize the process [8]. The International Accrediting Organization for Clinical Research (IAOCR) solves that problem and offers this service today, but it is not universally accepted or required like other forms of hospital accreditation. The major shortcoming of this effort is that it is yet again reliant on buy-in and process changes from all parties.

From these past efforts we see that for any sustained change to occur, the industry must first develop a shared comprehension of the problem and find value for all stakeholders in a proposed solution. The goals of this SEL Task Force were to identify problems in the process that might be new or unknown to all stakeholders, to raise awareness of those issues, and to identify potential solutions that could serve all stakeholders without requiring broad collaboration across the industry.

## 2 Methods

The SEL Task Force first defined the scope of the project and agreed on the definition of Site Feasibility. They reviewed the available literature on the issue. Next, they were divided into sub-groups of sites and sponsors/CROs to discuss current site feasibility processes and the challenges they experience with those processes. Each group also suggested desired outcomes from the SEL Task Force. The sub-groups reconvened and discussed the current processes and the challenges identified by each group. There was idea sharing between people in the same sub-group, and information sharing across sub-groups. As the challenges were discussed, some challenges were identified that not everyone in the SEL Task Force was aware of. Finally, the group aligned on the desired outputs, recognizing that it was important to share this information with the industry.

A collective idea that emerged was to create standards for data collection and to design an automation tool to reduce redundancy in the process. While this idea was appealing, it was quickly determined that a technical solution and process change for all stakeholders was an unreasonable goal for the SEL Task Force due to the issues discussed in the “Site Feasibility Challenges” section of this work.

A more attainable goal was to raise awareness across the industry of these new trends and share best practices to address the associated challenges. Members of the SEL Task Force committed to dissecting the challenges to identify the root causes and risks to all parties. By identifying root causes and risks they were also able to suggest best practices for avoiding these challenges. They agreed this was valuable since these challenges impact both “sides” of the industry, sites as well as Sponsors/CROs. The challenges, root causes and risks are presented here to help inform and drive change. Raising awareness now could minimize the impact of these trends or alter the trends in a meaningful way.

Finally, the group developed a checklist to help implement the best practices recommended here.

## 3 Results

The SEL Task Force identified three emerging challenges in the site feasibility process.

### 3.1 Challenge 1: Early initiation of site feasibility

A newly observed trend among the SEL Task Force members is that the Site Feasibility process is being initiated earlier, before the protocol is finalized. The group discussion suggests this is happening for two primary purposes: to gather site feedback for finalizing Study/Protocol Feasibility and to expedite the time to First Patient In (FPI). The result of this early initiation is that sites spend time on feasibility forms and visits for a protocol that is not finalized. Sponsors benefit from the site responses when finalizing and optimizing the protocol, but a completed protocol is required for all parties to accurately assess if potential sites are qualified, capable and interested in a study.

**Fig. 1:**
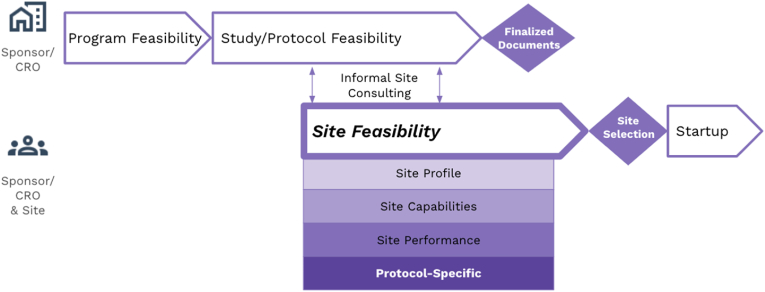
Early Initiation of Site Feasibility Fig. 1. Diagram showing one emerging trend in Site Feasibility, the early initiation of the Site Feasibility process. Site Feasibility starts during the Study/Protocol Feasibility process in order to get informal feedback on protocol design or to accelerate study startup. All phases of Site Feasibility overlap with Study/Protocol Feasibility.

Early initiation of Site Feasibility means that Study/Protocol Feasibility and all phases of Site Feasibility are overlapping, as shown in Fig. 1. To explain the issue, the SEL Task Force members developed a list of trending actions, as well as the associated impacts of proceeding with Site Feasibility under those conditions (Table 1). The overarching impact is that without complete protocol information sites are unable to confirm they have interest in the protocol, access to the target patient population, or the resources (people, facilities, equipment, and capabilities) to successfully support the trial. Often the finalized protocol information is not provided until much later in study startup. The downstream impact of this missing information is that sites are unable to accurately complete budget development processes (determine all the costs, qualifying clinical trial (QCT) determination, then either Medicare coverage analysis (MCA) or study reconciliation) and negotiate a budget fair to all parties, including the participants.

**Table 1:** List of trending actions and their impact on Site Feasibility.

Action/Item	Reason/Rationale	Impact	Best Practice
The full protocol is not sent until after selection; sites only receive slides or synopsis during feasibility.	- Protocol is not yet finalized but starting site feasibility to decrease time to FPI. - May be seeking input from experienced sites for protocol optimization; are willing to make protocol changes early in process (Especially true with smaller sponsors).	**Sites** are unable to make an informed decision on participation, provide accurate enrollment projections, determine compliance with standard of care treatment expectations, or determine if they have the resources available to meet all protocol requirements. **Sites** can't determine whether community or satellite locations can participate. **Sites** can only respond based on the provided information. A draft/synopsis is only valuable to determine initial site interest, not accurate site feasibility.	Site Feasibility should begin after the protocol is finalized or close to finalized. The full/final protocol, or a full draft or detailed synopsis to include mechanism of action (MOA), if applicable, and inclusion/exclusion (I/E) criteria as well as schedule of activities (SOA) should be shared.

Inclusion/Exclusion criteria are not finalized.	The protocol is not finalized but sponsors/CROs expect sites to estimate enrollment based on major I/E criteria to speed up the site selection process.	**Sites** are unable to accurately project enrollment estimates without knowing all I/E criteria.	**Sponsors** should finalize I/E criteria prior to site feasibility. The best practice is to leverage early stakeholder engagement to finalize I/E criteria.

Pharmacy manual is not available.	The manual is not finalized and/or the vendor has not been identified or the contract is not finalized.	**Sites** are unable to determine whether investigational pharmacy services have the resources, staff, or space to handle the investigational drug requirements.	**Sponsors** should share investigational product handling, stability and resource requirements during feasibility for sites to make an accurate assessment.

Detailed lab manual or operational requirements are not sent during feasibility (Ex: formulation requirements, sample collection, sample processing, equipment and shipping requirements).	- Lab manuals are not finalized and/or vendor contracts are not complete and draft information is not shared. - Vendors may be creating the manuals with a timeline for final completion close to FPI. - Sub-contracted labs for specialty biomarkers may further delay final lab manual completion.	**Sites** are unable to accurately assess if they have the personnel, equipment or ability to comply with the protocol requirements. **Sites/site networks** can't accurately identify which sites or community/satellite sites can participate without knowing the full capabilities required.	**Sponsors** should send all detailed **DRAFT** material (sample types, equipment and requirements) during feasibility. **Sites** should ask for the draft or final manuals to make an accurate assessment of their ability to conduct the protocol. **Sponsors/CROs** should expect and allow for rapid budget renegotiation of budget if draft materials are not shared.

Detailed information on central labs is not shared during feasibility (Ex: sample shipping details, processing time, results delivery timelines, on-site banking, short-term requirements, etc.). This is especially detrimental if the central labs are involved in eligibility assessments.	Central lab role/function is not finalized or the vendor contract is not complete.	**Sites** are unable to plan for and may later struggle to comply with protocol requirements due to lab processing times and shipping constraints they were not aware of, which impacts visit scheduling and staffing requirements. **Sponsors** may have unrealistic expectations related to a protocol's schedule of assessments and/or assumed participant decisions. **Sponsors/CROs** are unable to filter out sites that cannot meet the lab requirements, resulting in unnecessary expenditure of time and effort.	**Sponsors/CROs** should engage vendors earlier. **Sponsors/CROs** should seek operational feedback from sites (as an early stakeholder engagement activity) to confirm and finalize lab details. All details and vendor contracts should be finalized/executed prior to the initiation of site feasibility. **Sponsors/CROs** should allow local labs for eligibility assessments. **Sponsors/CROs** should plan for vendor limitations and include mitigation options in the contract.

Imaging requirements are not shared at the time of feasibility (Ex: required qualification scans, calibration requirements, staff training, healthy volunteer sample scans, etc.).	Imaging manuals are not finalized and/or vendor contracts are not complete and draft information is not shared.	**Sites** are not able to accurately assess their timelines to complete the necessary logistics or additional requirements needed to participate (Ex: additional ICF for healthy volunteers).	**Sponsors/CROs** should send all detailed **DRAFT** imaging material and communicate pre-activation requirements during feasibility.

Data points to be collected are not all included in the protocol or lab manuals (There are required data points only found in the case reports forms (CRFs)).	Sponsor/CRO data science teams add detailed data points during CRF writing, which often occurs later in the process than site feasibility.	**Sites** cannot assess if they have all the required equipment or processes in place to adhere to the protocol and meet all data collection requirements.	**Sponsors/CROs** should ensure that ALL data to be collected is mentioned in the protocol or manuals or that draft CRFs are shared.

CROs/Sponsors are not transparent about the timeline and status of the study (FDA submission status, protocol completeness, planned amendments, etc).	Desire to select sites as early as possible to decrease timelines, don't want to demotivate or lose sites when there are delays.	**Sites** are unable to prioritize their study pipeline and may decline studies they could do later or accept studies that end up competing with others due to unclear timelines. **Sites** work on feasibility at the risk of the study not being approved by the FDA. **CROs** are unable to proactively plan the utilization of personnel resources.	**Sites** should ask Sponsors/CROs about study status. With a list of specific questions, they may be able to gauge the study status to better plan internally and more accurately respond to feasibility questions during the selection process.

CROs/Sponsors are not transparent about enrollment status for studies that are already enrolling.	Enrollment and timelines change quickly and there is not a mechanism to ensure updates are provided during feasibility and startup.	**Sites** can't accurately estimate enrollment projections and timelines without knowing the current enrollment status of the study and each arm or cohort, as applicable.	**Sponsors/CROs** should provide regular updates on enrollment to sites in feasibility and startup.

Realistic draft budget is not shared during the feasibility process.	Sponsors/CROs don't want to share too many details with sites before they are selected.	**Sites** can't determine if there are major budgetary concerns that will cause delays and/or render the study cost-prohibitive during the feasibility process.	**Sponsors/CROs** should share a draft budget or any major budget constraints/limits prior to site selection.

Sites do not accurately estimate enrollment projections.	Not all sites have good data and tools for estimating enrollment activity. Sites are often basing estimates on only partial protocol information (as mentioned above).	**Sponsors/CROs** can't effectively gauge participation for the study, leading to inaccurate project timelines, resource needs, and overall cost for all parties.	**Sites** should be as accurate as possible with enrollment projections, using tools and data to estimate participant populations.

Sponsors/CROs are not responsive to site questions during feasibility.	- CROs cause delays in collecting and asking questions to the sponsor and relaying them to sites. - Sites do not always have a clear communication channel to the Sponsor, especially when a CRO is involved.	**Sites** are delayed in responding or are unable to respond when they can't get timely and accurate responses to questions.	**Sponsors/CROs** should establish clear communication channels and a plan for timely responses.

Sites are not responsive to Sponsor/CRO questions during feasibility.	Sites may have staff turnover, may be overwhelmed with study activities, or may decide not to complete the feasibility process. Sites may not be able to address feasibility questions with information as provided by sponsor/CRO or be awaiting responses from supporting service areas.	**Sponsors/CROs** can't accurately assess potential site lists or plan for startup timelines when sites do not respond. Sites may be replaced by more responsive sites.	Sites should establish clear communication channels and a plan for timely responses. Sites should be clear regarding the reason for delay in answering feasibility questions. Sites should decline if they are not interested.

The feasibility meeting burden on CRO and site staff is too high and involves redundant work.	Sponsors/CROs want to ensure sites are qualified and have all documentation to show that sites were thoroughly evaluated.	**Sites and CROs** waste time and resources on redundant meetings.	**Sponsors/CROs** should prioritize key PSV deliverables and work to reduce the meeting burden on sites by not repeating questions asked earlier in the feasibility process. Leveraging accurate site profile information could support this best practice.

A key finding of the SEL Task Force is that many sites are either unacquainted with this trend or have not experienced it yet, and the potential impact remains unnoticed by sponsors, or is attributed to site startup delays rather than a systemic problem with the feasibility process. To help further explain the problem and impact, the SEL Task Force members shared the following examples.

#### 3.1.1 Real world examples

**Example 1.**

**Impact:** This new study requirement meant that the IT and EHR team had to get involved, privacy language had to be added to the informed consent form (ICF), a Data Use Agreement had to be put in place and the budget had to be re-negotiated to account for the new work.
**Result**: Study Startup Delay.

**Example 2.**

**Action:** A site found out at the SIV that the pharmacokinetic (PK) lab processing required a refrigerated centrifuge. This detail and the lab manual were not shared during the feasibility process, and this is not typical for PK lab samples.
**Impact:** Not all sites have this equipment. The site had to find a department in the hospital that had this resource and was willing to allow the study team to use it. The study staff had to travel to the alternate department and stay with the sample for immediate aliquoting. This required more staff resources than planned for the study, especially on days where several samples were drawn.
**Result**: Site Budget Renegotiation, Risk of Protocol Deviations.

**Example 3.**

**Action:** During feasibility the site confirmed that no local labs were required per protocol. At the SIV the site was informed that for safety reasons, in certain situations they were required to run lab tests with the local lab in addition to sending samples to the central lab.
**Impact:** The site had to work with the local lab to add them to the study, which required additional training, updates to regulatory documents, and contract and budget amendments.
**Result**: Study Startup Delay, Risk of Protocol Deviations.

**Example 4.**

**Action:** After the study contract negotiations, PSSV, and SIV, the sponsor said that a Neurologist was required as the PI and the site could not use the previously approved Internal Medicine PI. This information was never noted during feasibility, contract negotiations, or review of the initial PI's curriculum vitae (CV).
**Impact:** The site was unable to find a Neurologist willing to be PI.
**Result**: Site Dropout post-SIV (Startup costs were still owed to this site, additional time and cost to replace this site).

**Example 5.**

**Action:** During the SIV the site learned that a particular lab test was required to be run onsite. This information was not shared during the feasibility process.
**Impact:** The site was unable to use several of the planned satellite sites who did not have the right equipment. Some of those satellite sites were planned to contribute heavily to enrollment. Potential patients were not willing to travel to the main hospital for visits.
**Result**: Wasted effort and cost for satellite site startup, Inaccurate enrollment projections.

**Example 6.**

**Action:** During the SIV the site learned that the central lab had specific processing requirements and shipping restrictions (samples could not be shipped on Thursday or Friday or stored onsite). This information was not shared during the feasibility process.
**Impact:** The site was unable to schedule patient visits on Wednesday afternoon, Thursday or Friday, which conflicted with the PI office schedule and caused staff resource constraints.
**Result**: Risk of Protocol Deviations.

**Example 7.**

**Action:** The site requested the pharmacy and lab manuals during feasibility, but only the lab manual was available. The sponsor stated that the investigational product (IP) needed refrigeration. Not all the site facilities have separate refrigerators for IP, but they were able to find space in an inpatient pharmacy. At the SIV during the pharmacy tour the site was told that the IP had to be formulated in a hazardous compound hood.
**Impact:** The site did not have a hazardous compound hood and was forced to withdraw from the study after all startup work was completed.
**Result**: Wasted startup effort and cost, Site Dropout post-SIV.

### 3.2 Challenge 2: Premature engagement of sites in feasibility process by CROs

The second challenge identified by the group is the practice of CROs initiating the Site Feasibility process before securing the study contract from the sponsor. This process is most likely driven by the desire to have a ready set of highly qualified sites to present to the sponsor as part of their bid for the study. Alternatively, it serves to jumpstart the site selection process at the earliest possible moment once the study has been officially awarded. The key finding in this case was that some sites and sponsors appear to be unaware that this is taking place, suggesting a lack of communication and transparency within the current system.

### 3.3 Challenge 3: The crucial role of clear and timely communication

The third challenge is that there is a lack of clear and timely communication across stakeholders in the feasibility process, which can adversely affect the process, leading to inaccurate commitments, resource misallocation, and overall inefficiencies in the trial process.

As these challenges were identified, the group was able to make recommendations for the industry.

### 3.4 Recommendation 1: Site feedback should occur via formal consulting during Study/Protocol Feasibility

**Fig. 2:**
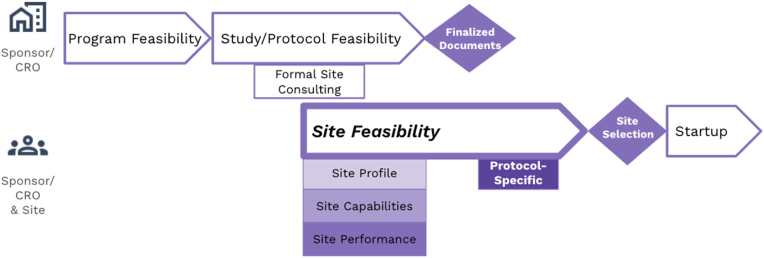
Recommended Feasibility Process Fig. 2. Diagram showing SEL Task Force recommendations for the feasibility process. Sponsors and CROs should get site feedback for Study/Protocol Feasibility via formal consulting arrangements. Site Feasibility profile, capability and performance information can be collected and assessed prior to the protocol documents being finalized. The protocol-specific feasibility assessment should occur only after all protocol documents have been finalized. This proposed order of events will reduce burden on site time and resources and ensure a more accurate assessment of protocol-specific requirements.

The first recommendation from the SEL Task Force is that clear boundaries should be set between Study/Protocol Feasibility and Site Feasibility. There was clear agreement that sites ***should*** be providing feedback during the Study/Protocol Feasibility process, before Site Feasibility. This recommendation is specifically stated in section 3.1.3 of the recently updated ICH Harmonised Guideline for Good Clinical Practice, R3 [12] as part of the Trial Design process, and is also mentioned in the CTTI Quality By Design document [13]. However, this feedback should occur via formal consulting agreements, not as part of the Site Feasibility process (Fig. 2). The Site Feasibility process should be used for identifying and assessing potential sites for a specific study. A completed protocol is required for all parties to accurately assess if potential sites are qualified and interested in conducting a study.

Input from sites should occur during protocol design via an early stakeholder engagement framework and should include operational and scientific feedback. The operational details of the protocol are often overlooked and many assumptions are made about the ability to conduct protocols. Sites are eager and willing to consult on operational details of protocol design because they want protocols that are clinically reasonable and operationally viable. In order for their feedback to be actionable, sites need to be engaged earlier in the protocol writing process. Furthermore, the SEL Task Force consensus was that protocol feedback should be a paid consulting opportunity.

Creating clear separation between the two phases, or at least being transparent about the stages of the process will prevent delays and disruptions. Transparency throughout the process, from all stakeholders, is critical to success. If critical details are not yet finalized when the site feasibility process begins, and the site is not informed, it could have a significant downstream impact.

### 3.5 Recommendation 2: Site feasibility should be conducted in phases to improve operational efficiency

A second recommendation of the SEL Task Force is that the Site Feasibility process should also be divided into phases (Profile, Capability, Performance and Protocol-Specific). Sponsors should consider separating these phases as ways of engaging with sites sooner without impacting startup and recruitment timelines later (Fig. 2). For example, by collecting site profile, capability and performance data, which is unlikely to change much in a short time period, early in the process, they can engage sites and start the process before the protocol (and associated documents such as lab manuals) are finalized. The protocol-specific feasibility phase can occur after the protocol is finalized, allowing sites to focus on the new questions with less redundant work. It would be even more ideal to have real-time access to current site profile, capability and performance data from a large number of sites. The Shared Investigator Platform (SIP) was intended to provide this functionality, but in the SEL Task Force's experience, this has not materialized. By having this profile, capability and performance data, sponsors can identify a more targeted list of sites to engage for the fourth phase of Site Feasibility, protocol-specific assessments. With a targeted list of sites and real-time/current data on capabilities and performance, Sponsors/CROs would also be able to reduce the questions asked to sites, streamlining communication, reducing redundant work, and focusing on the critical details required for sites to make an accurate assessment of their ability to execute a protocol. As mentioned, AQC is leading the industry by taking the steps to develop tools that can more effectively facilitate the process. However, these tools must be more widely available and uniformly adopted by sites, sponsors and CROs to create a basis for further improvements.

### 3.6 Best practices for implementing the recommendations

For each action and risk seen in the current process, the SEL Task Force identified a best practice in line with these recommendations (Table 1). Of note, many best practice recommendations suggest sending the draft version of an item. The SEL Task Force members strongly preferred to receive the final document, but agreed to the draft as a compromise since it lets sites proceed with a little more confidence in answering feasibility questions. It should be clear, however, that sites can only respond based on the provided information. A draft/synopsis is not always enough to ensure an accurate site feasibility response.

The best practices identified in Table 1 can be distilled into a checklist of minimum necessary documents required before Sponsors/CROs begin the Protocol-specific Site Feasibility process (Table 2). Before any party begins the process, these critical path items should be available. For sponsors, this will reduce inaccurate site responses, and for sites this will result in a more efficient process later during site startup. For all parties, this will result in the least risk and the most optimal outcome. Although it might delay the beginning of the site selection and feasibility process, the reduction in rework and delays later will benefit all parties.

**Table 2:** List of required materials to initiate Protocol-specific Site Feasibility.

Status	Material
***DOCUMENTS***	
□	Finalized Protocol
□	Lab Manuals (Draft or Final)
□	Pharmacy manual (Draft or Final)
□	Imaging manual/requirements (Draft or Final)
□	Non-redacted FDA approval letter (for IDE, IND studies)
□	Finalized CRFs
□	eCRF completion guidelines
□	Central lab shipping requirements
□	Equipment List (Draft or Final)
□	Budget template (Draft or Final)
□	Operational manuals
□	NCT number
***COMMUNICATIONS***	
□	Site Point of Contact Email & Phone Number
□	Sponsor Point of Contact Email & Phone Number
□	CRO Point of Contact Email & Phone Number
□	Communication Plan
***TOOLS DISCLOSED***	
□	List of all vendors and systems being used
□	EDC
□	Regulatory
□	Source
□	Recruitment
□	Consent
□	Inventory Management
□	Other: __________
***MISCELLANEOUS***	
□	Pilot/early phase data
□	Projected timelines for all parties

Note that at some sites that have research oversight committees or scientific review committees, it may be better to start the protocol-specific feasibility process before all these documents are available since that process can add up to a month to the process. Knowing site profile information would allow a sponsor to recognize when this might be the case and deviate from this recommendation with a better outcome.

Once the risks of the current site feasibility process were understood and best practices were identified, the SEL Task Force was able to provide specific actionable steps that stakeholders can follow to implement the suggested improvements. These simple guides make the recommendations more practical and increase the likelihood of their adoption.

### 3.7 Guide for Sponsors/CROs

● Engage sites in protocol optimization conversations with early stakeholder engagement meetings and consulting opportunities during the Study/Protocol Feasibility stage.
● Begin Site Feasibility early in the study development process with ONLY the Site Profile and Site Capabilities phases.
  ○ Use a system that provides up-to-date site data to speed up the process and reduce the site workload.
  ○ Share within your organization; many times multiple stakeholders from the SAME organization are reaching out to the site asking the same questions.
● Ensure all critical path items are complete (or drafted) BEFORE starting the protocol-specific assessment phase of Site Feasibility.
  ○ Refer to the Site Feasibility Checklist provided here (Table 2), or
  ○ Consider the impact/risk of not including an item, as described in Table 1.
  ○ Note that final documents are required for sites to accurately assess their ability to meet protocol requirements and for their feasibility assessment to be valid.

### 3.8 Guide for sites

● Understand and document the steps of your feasibility and start-up processes to allow for consistency and understanding of how the site works. This is useful for internal and external stakeholders.
  ○ Identify and remove internal bottlenecks wherever possible.
  ○ Develop a standardized approach which incorporates the tools required to allow for automation and efficiency.
  ○ Understand how this process is impacted by sponsor/CRO processes, since Sponsor/CRO variability does impact site efficiencies.
  ○ Request and review all sponsor materials when received.
  ○ Inventory sponsor documents. Immediately request items which are missing, in draft or not in final form from sponsor/CRO.
  ○ Site standardization for feasibility and startup results in decreased errors and rework due to omissions.
● Communicate the process and expectations clearly to the Sponsor/CRO.
  ○ Provide sponsor/CRO with the site process overview when starting the feasibility process. Be clear on expectations and steps of the process.
  ○ Confirm receipt and understanding of sponsor/CRO expectations and responsibilities in site feasibility and start-up processes.
  ○ Identify realistic timelines for all steps in the process to give the Sponsor/CRO clarity of what they can expect from the site. Communicate timeline changes real-time, including the reason for any change.
  ○ Proactively request a meeting with Sponsor/CRO when experiencing difficulty meeting timelines for submissions or negotiations.
  ○ Promptly respond to Sponsor/CRO requests, especially if it is a reminder that the site is waiting on them to proceed.
  ○ Rapidly escalate issues to the sponsor when the site feels CRO is being non-responsive; this is equivalent to the sponsor/CRO escalating issues to the PI when there is a concern or delay.

## 4 Discussion

As anticipated, the group cited numerous examples of challenges referenced in the ASCO paper [2], namely redundancy and lack of standardization, which result in inefficiency such that the process consumes valuable site resources. The three unexpected findings that arose from the discussion were unique. From the sites’ perspective, these trends are either newly emerging or worsening, and are leading to subsequent disruptions and significant delays during startup. Amongst the sponsor and CRO participants, there was mixed awareness of these trends and their consequences. Note that there is wide variability in the size, available resources, and experience level of Sponsors, CROs and sites across the industry, as well as across therapy areas and disease states, which means that not all findings apply to all groups. Nevertheless, it is important to share trends observed in the industry before they become widespread.

### 4.1 Challenge 1: Early initiation of site feasibility: implications and unforeseen consequences

When the Site Feasibility process is being initiated earlier to utilize site feedback for finalizing Study/Protocol Feasibility, sponsors are effectively engaging the earliest potential sites, typically experienced research centers and/or sites with Key Opinion Leaders (KOLs), to gain insights for Protocol Optimization. The feasibility evaluations serve as a conduit to refine the protocol based on the input from these sites regarding aspects like procedure requirements, treatment details, inclusion/exclusion criteria, and participant burden. This feedback is obtained through queries from the site and/or discussions with the site and PI during the feasibility phase or at the site visit (Pre-selection site visit/Site qualification visit).

When the Site Feasibility process is being initiated earlier to expedite the time to First Patient In (FPI), Sponsors and CROs aim to reduce overall study timelines. A primary step towards this goal involves quicker identification and selection of sites. Given that many sponsors frequently collaborate with the same sites, it may seem obvious to begin the feasibility process as early as possible. However, Site Feasibility is often beginning before all the protocol, lab, and vendor specifics are finalized.

In both situations, the lack of precise details and/or the subsequent additions/changes to the protocol and supporting documents can invalidate a site's responses to the feasibility questions. For example, specific requirements and details about sample processing, storage necessities, central lab locations, vendor requirements, and equipment often determine a site's capability to participate or involve satellite facilities. When such details emerge much later during startup, sites might be unable to comply with the requirements or may need to revise IRB approvals, budgets, contracts, and facility plans. This is often not discovered until after selection, or even as late as at the Site Initiation Visit, and can dramatically impact a site's ability to participate in the study. In many cases, the result is disruptions and delays during the startup phase. Protocol details that have not been shared cannot be accounted for during feasibility; transparency about such uncertainties should be shared in a timely manner to avoid negative impacts.

The group noted that this does not happen with all Sponsors and CROs and not all sites are targeted for this earlier feasibility process. A final component of this challenge is that sites are typically not compensated for feasibility assessment work, and the uncertainty in this accelerated process means a site is investing even more resources than they would for a normal site feasibility process.

### 4.2 Challenge 2: Premature engagement of sites in feasibility process by CROs

With the emerging practice of CROs initiating the Site Feasibility process even before securing the study contract from the sponsor, sites often find themselves involved in what appears to be a normal feasibility process, with no awareness of the additional layer of uncertainty. Sites are not typically told that they are only getting partial protocol information. They may be unaware that the opportunity for the study itself is far from certain, as the contract has yet to be awarded to the CRO. This preemptive engagement with sites is a considerable commitment of resources and time for both sites and CROs, leading to waste if the contract is not awarded, and inaccuracies in the long run due to incomplete protocol information.

Interestingly, this emerging trend seems to have flown under the radar for most sites and sponsors. It is imperative to bring this to the attention of all involved parties to prompt a reevaluation of current practices and to mitigate any adverse effects on the feasibility process.

### 4.3 Challenge 3: The crucial role of clear and timely communication

Effective communication sits at the heart of a successful feasibility process. However, the occurrence of unclear or delayed communication, or difficulties in bridging gaps between different parties, can substantially hinder the process. Critical information about realistic timelines, desired milestones, actual statuses, knowns and unknowns is paramount to effective resource optimization at sponsors, CROs and sites. This impacts appropriate staff allocation, scheduling of study activities, and an accurate understanding of the study pipeline and portfolio to ensure reliable commitments to study enrollment numbers.

Interestingly, this issue impacts all parties in the process. If sponsors or CROs are not sharing crucial information then sites and/or CROs can't be confident in their resource planning. If sites are not transparent about their timelines or don't offer highly accurate responses to enrollment planning questions, then sponsors and CROs can't allocate resources appropriately. Tackling this tri-directional challenge is paramount to making progress in the industry.

The discussion in the SEL Task Force indicated a consensus that these communication-related challenges tend to be amplified when all three parties - the Sponsor, CRO, and Site - are involved. The potential for miscommunication or misunderstanding increases with each additional participant, necessitating a deliberate and coordinated approach to communication.

To illustrate, consider the area of transparency in site selection: Sites often express a desire for clear and honest feedback regarding their non-selection for a specific study. Having a transparent understanding of the reasons behind their exclusion can empower sites to improve their performance and enhance their prospects for future participation. However, this valuable insight can only be gained through open, honest, and timely communication from the sponsors and CROs.

## 5 Conclusion

The set of challenges identified by this SEL Task Force highlight the risks of navigating the feasibility process without considering the impact on all stakeholders. This process was explored because of the extreme burden and repetitive work the Site Feasibility process creates for sites. However, the actual challenges identified have far greater impact on clinical trials than has ever been discussed. The downstream impact on startup timelines and recruitment/enrollment goals is difficult to quantify but clearly meaningful. By bringing awareness and advocating for the industry to remediate these issues now, all stakeholders will benefit. Positive change in this area can result in faster startup times, reduced work, burden and cost for all stakeholders, improved participant recruitment and retention, and improved data quality.

This paper has shed light on a number of emerging issues; however, it is critical to acknowledge that the clinical trials landscape is vast and complex. The specific issues encountered may vary, influenced by factors such as the nature of the trial, the therapeutic area, and the range of stakeholders involved. The SEL Task Force is aware that not all of these issues will apply in all cases.

The goal of the SEL Task Force was not just to identify problems, but to inspire innovation and proactive improvements that can serve all stakeholders without requiring broad collaboration across the industry.

With this mindset, the SEL Task Force hopes to avoid the failures of past efforts in this area. Instead of proposing standardized solutions, these findings allow all organizations to evaluate their own processes and implement collaborative solutions that deliver value.

We hope that this paper serves as a catalyst for action and invites further discussion on the subject, encouraging the industry to move beyond recognition of the issues and towards the implementation of practical solutions. Through sustained efforts and dedication, we have the potential to shape a future where the feasibility process is not a hurdle, but a facilitator in the pursuit of successful clinical trials.

## Funding sources

This research did not receive any specific grant from funding agencies in the public, commercial, or not-for-profit sectors. Florence Healthcare employees manage the Site Enablement League (SEL). All members of the SEL were invited to participate in this SEL Task Force; participation was optional. Members of the SEL and task force are not paid for their contributions or their time.

## CRediT authorship contribution statement

**Beau Bruneau:** Conceptualization, Methodology, Visualization, Writing – review & editing. **Kristin Surdam:** Conceptualization, Methodology, Writing – review & editing. **Amy Bland:** Conceptualization, Methodology, Writing – review & editing. **Amy Krueger:** Conceptualization, Methodology. **Andrew Wise:** Conceptualization, Methodology, Writing – review & editing. **Ani Cotarlan:** Conceptualization. **Asher Leviton:** Conceptualization. **Elena Jouravleva:** Conceptualization, Methodology, Writing – review & editing. **Grace Fitzgerald:** Conceptualization, Methodology. **Heather N. Frost:** Conceptualization, Methodology. **Honora F. Cutler:** Conceptualization, Methodology, Writing – review & editing. **Joshua Buddle:** Conceptualization, Methodology. **Luis G. Diaz:** Conceptualization, Methodology, Writing – review & editing. **Michele Cohen:** Conceptualization, Methodology, Writing – review & editing. **Nancy A. Sacco:** Conceptualization, Methodology, Writing – review & editing. **Ryan Washington:** Conceptualization, Methodology, Writing – review & editing. **Susan Mauermann:** Conceptualization, Methodology, Writing – review & editing. **Victor Chen:** Conceptualization, Methodology, Writing – review & editing. **Andrea Bastek:** Conceptualization, Methodology, Writing – original draft, Writing – review & editing, Visualization.

## Declaration of competing interest

The authors declare the following financial interests/personal relationships which may be considered as potential competing interests: Three authors are employees of Florence Healthcare, a technology vendor in the clinical trial industry.
